# Ten simple rules for hitting a home run with your elevator pitch

**DOI:** 10.1371/journal.pcbi.1008756

**Published:** 2021-03-18

**Authors:** Whitney Rachel Morgan, Erik Scott Wright

**Affiliations:** 1 College of Law, West Virginia University, Morgantown, West Virginia, United States of America; 2 Department of Biomedical Informatics, University of Pittsburgh, Pittsburgh, Pennsylvania, United States of America; Dassault Systemes BIOVIA, UNITED STATES

## Introduction

Many academics feel ill-prepared for a world where selling yourself is part of the game. Maybe you did a sacrificial bunt with your social skills way back in childhood to chase more brainy pursuits. Or perhaps you never bothered studying the intricacies of pitching because it is outside your field of expertise. Now you have found yourself in the major league: meeting a visiting professor, drafting a personal statement, giving a poster presentation, interviewing for a job, or advertising your work on Twitter. A bit longer than the half second a baseball travels from mound to mitt, pitching yourself effectively can spread the word about your work and open doors for your future academic success in only a few minutes. Beyond the elevator, you never know what opportunities life will throw your way, so it is best to be prepared with these 10 simple rules.

### Rule 1: Define success

Before you can succeed, you must identify what it is you want to achieve. Pitches fall into 1 of 3 categories, depending on your goal: (i) creating connection with your audience in order to establish shared values or build communion, (ii) encouraging collaboration by getting the audience to take action or bring them to your side, or (iii) generating awareness through education and being informative [1]. Like any good pitcher, you must decide on the type of pitch you will throw before your windup. Ask yourself, “What would success look like in this situation?” Knowing your goal also helps to establish the best mode of persuasion. Aristotle defined 3 types of appeals as ethos (to credibility), pathos (to emotion), and logos (to logic) [2]. Academics often construct logical appeals, purposefully devoid of emotion, and take their credibility for granted. However, people tend to buy into appeals based on emotion or credibility and later justify their decision based on logic [3]. Good pitches will make all 3 types of appeals, albeit in varying proportions, but focus on a single type of pitch from the outset.

### Rule 2: Make the introduction

The windup can be the most important and intimidating part of the pitch. People form their initial opinion about a person within only a few seconds, and this opinion determines much of what they think about later appeals. But there is no need to stress. Remember that you are a natural pitcher and have been making pitches through your emails and conversations for years [4]. Moreover, your audience is secretly cheering for you to succeed, as it may benefit them too [5]. Most people have many competing interests for their time, so a good way to begin is by asking for the amount of commitment you need upfront. For example, at a poster session, you could ask someone glancing at your poster, “Could I take three minutes to explain the discoveries I made in this project?” This relieves your audience’s anxiety about time commitment and breaks the ice for you to introduce yourself. Be sure to provide personal context and establish credibility with a quick sentence about yourself. Including specific imagery can make it easier for your audience to connect (“I am from Pittsburgh, which is home to one of the best ballparks in America because of its panoramic views of the city skyline.”). You might also consider sparking your audience’s curiosity with a rhetorical question. For example, you could say, “Did you know the terminal velocity of a baseball is 100 miles per hour? Imagine if every pitcher could learn to throw faster than terminal velocity.” Asking a rhetorical question followed by a sentence starting with “Imagine” is a great way to avoid appearing out of left field [6].

### Rule 3: Embody your pitch

Now that you have the bases loaded, it is critical to remember the other facets of communication that collectively represent a huge fraction of what is communicated. Enter your pitching stance by standing upright with your torso turned toward the other person to physically show engagement. People naturally respect those whose posture commands respect. Making eye contact can help establish connection and is easier to accomplish if you have already planned and practiced your pitch. Use your hands to demonstrate what you are saying and tune your body language. Consider adding props or a video to support your case. It is especially important to speak as if having a one-on-one conversation even if multiple people are present. To connect with a larger audience, shift your attention across the group but focus on maintaining a conversational style of communication. Great communicators carefully place pauses to interject space around their key points and slow down their speaking [7]. Pauses also help to give the audience time to comprehend and respond with clarifying questions. Getting your pitch right (not baseball, the other one) does a lot to set the overall tone of the encounter. Just like a baseball pitcher might use a changeup to keep the batter on their toes, using a dynamic voice (tone, pace, and volume) will energize your pitch.

### Rule 4: Tailor to your audience

The most critical, and perhaps most challenging, component of the elevator pitch is to appeal to your audience’s motivations. In this way, you must be a servant of your audience and not of yourself, because you will miss the strike zone if you only serve your own ends. The most successful pitches are the ones where the catcher feels engaged to the point where they are collaborating with the pitch [6]. To achieve this sort of win-win situation, you have to know a thing or two about your catcher. Ideally, you would do your homework about their interests, position, and accomplishments ahead of meeting. This is particularly true for written communication such as personal statements, cover letters, and emails where there is an opportunity to research your audience in advance. If you do not get a head start, you can look around for signs that will help you read your catcher, such as paying attention to what’s on their nametag (at a conference) or bookshelves (in an office) [8]. Here especially is where asking questions and being a good listener can pay dividends by helping you establish favorability and tune your pitch to your audience.

### Rule 5: Tell a story

Having reached the heart of your pitch, you have around a minute to convey the essence of your appeal. The best way to hit the sweet spot is through a story that conveys your authentic excitement. The right story can make both you and your audience come alive with enthusiasm. To find this story, try to recall what it was like to learn for the first time what you are now pitching—the “Eureka!” moment. Put yourself back in that scene, and then bring your audience there with you. Where possible, focus on a single person in your story because people have an easier time thinking about individuals than groups. Ideally, you could reenact at least one real-world example of what it is you want to communicate. The core of the pitch should provide sufficient evidence but avoid seeming like a big data dump. More specifically, great pitches focus on the outcomes rather than the process, saving the details for later [8]. This is the point where you must have the goods to deliver. No amount of buzzwords, jargon, or Yogi Berra quotes can hide a lack of content.

### Rule 6: Focus on “why”

As you finish your story, it is time to keep up your winning streak by explicitly stating the “why”: Why what you are pitching is important to you, to your audience (see Rule 4: Tailor to your audience), and to the world in general. The “why” is your money pitch, what makes your audience feel moved to the point of taking action [9]. This is the point in the pitch where you establish how you want your audience to feel and impart the perspective that they will take with them after your pitch is over. One way to accomplish this is to teach people how to think about the story (see Rule 5: Tell a story) by starting a sentence with, “The way I think about this is…” [10]. Do not assume that the “why” is obvious to everyone else. Clearly conveying why what you are pitching is compelling or changed your thinking will help you reach a more diverse audience with a wide variety of experiences and backgrounds. You may even find yourself needing to rediscover the “why” that motivated you in the first place. Bringing the “why” to the forefront of your pitch will make it more influential and may very well make the difference between striking out and the hall of fame.

### Rule 7: Keep it positive

At this point, you have entered the seventh-inning stretch, and it is important to keep up your momentum by exuding passion and positivity. Remember to smile as you convey the positives underlying your case. Specifically focus on what solutions and opportunities you are providing. This is a good time to add some light humor, but steer clear of any foul balls or negativity. Oftentimes, academics are critical thinkers trained to hone in on the pitfalls or downsides of what is being presented. However, during a short pitch, it is important to bear in mind that people react much more strongly to bad than to good [11], so fill your first impression with the positives and amplify the good. Positivity is infectious and an exuberant personality will undoubtedly leave a better impression.

### Rule 8: Be memorable

Now it is the perfect time to bring in the closer. Here your goal should be for the person walking away to want to share what you said with others, which is the best way to spread the word about what you are pitching [12]. The challenge is to ensure that your audience understands and appreciates your pitch well enough to retell it [3]. To achieve this high aim, you must distill what you want your audience to remember down to just a few memorable words. After all, communication is what the person takes away, not what you said. Try to recall previous presentations that you have attended in the past year. Likely many of them are difficult to remember. But you can probably remember the best ones because of specific moments that stuck. One way to generate these moments is by developing sticky phrases with rhymes or alliteration that summarize your pitch, such as “good pitches yield riches” or “perfect pitches possess positivity.” Advertisers employ these short phrases regularly to embed their product in your memory. Repeating your own memorable phrase will help convert your audience into a fan base.

### Rule 9: Make the ask and follow up

With any pitch, you need to prepare an exit plan for the ninth inning. The pitch is often the first word but rarely is it the last [4]. In concluding your pitch, be direct in your effort to initiate the next (hopefully longer) conversation that you established as your goal (see Rule 1: Define success). Convey what you are asking of your audience: connect again, spread the word, collaborate, read your work, be a mentor, etc. Ideally, this includes details for a longer dialogue at a later date. It is best to give specific ways to get in touch again, such as, “I’ll be the one in the baseball cap at our next break; let’s talk some more.” If such an opportunity does not exist, consider asking for your audience’s contact information so you can reconnect with them again quickly. Expressing gratitude for the meeting in a follow-up email is a great way to reinforce your pitch and tilt the odds of success in your favor.

### Rule 10: Practice, practice, practice

The game’s the same no matter the field: Practice makes perfect. The brevity of an elevator pitch demands discipline and forces clarity. Rehearsing your pitch with others will help you to refine and optimize your wording. Practice your pitch on friends and colleagues, but be sure to encourage them to throw you some curveballs because you need to be prepared for heavy hitters. Getting feedback is essential to determine the parts of your pitch that are successful and those that are off base. Feedback may also reveal what your audience will remember afterwards (see Rule 8: Be memorable). You will know you are ready when your practice audience asks lots of questions [8]. But to get there, you will need practice, practice, and more practice. Try recording your pitch, and pay attention not only to your substance but also to your delivery (see Rule 3: Embody your pitch). Just like in baseball, great pitchers are made and not born.

## Conclusions

The short format of the elevator pitch makes it challenging to accurately convey a message while avoiding generalities and being transparent about limitations. The process of preparing in advance goes a long way toward making a concise pitch as complete and effective as possible. With enough thought, it is possible to construct a pitch that covers all the bases, engages your audience, and hits on the main points. There are many examples available to those hoping to master the art of pitching, including short TED talks, lightning talks on YouTube, and even the television show Shark Tank. Pitching is ubiquitous, so keep an eye out for opportunities to practice your pitch, and remember to ask for feedback when the opportunity arises. While great pitches are challenging to deliver, being well-prepared can help you knock it out of the park.
